# General hospital staff worries, perceived sufficiency of information and associated psychological distress during the A/H1N1 influenza pandemic

**DOI:** 10.1186/1471-2334-10-322

**Published:** 2010-11-09

**Authors:** Panagiota Goulia, Christos Mantas, Danai Dimitroula, Dimitrios Mantis, Thomas Hyphantis

**Affiliations:** 1 Consultation-Liaison Psychiatry Unit, Department of Psychiatry, Medical School, University of Ioannina, Greece

## Abstract

**Background:**

Health care workers (HCWs) presented frequent concerns regarding their health and their families' health and high levels of psychological distress during previous disease outbreaks, such as the SARS outbreak, which was associated with social isolation and intentional absenteeism. We aimed to assess HCWs concerns and anxiety, perceived sufficiency of information, and intended behavior during the recent A/H1N1 influenza pandemic and their associations with psychological distress.

**Method:**

Between September 1^st ^and 30^th^, 2009, 469 health-care workers (HCWs) of a tertiary teaching hospital completed a 20-item questionnaire regarding concerns and worries about the new A/H1N1 influenza pandemic, along with Cassileth's Information Styles Questionnaire (part-I) and the GHQ-28.

**Results:**

More than half of the present study's HCWs (56.7%) reported they were worried about the A/H1N1 influenza pandemic, their degree of anxiety being moderately high (median 6/9). The most frequent concern was infection of family and friends and the health consequences of the disease (54.9%). The perceived risk of being infected was considered moderately high (median 6/9). Few HCWs (6.6%) had restricted their social contacts and fewer (3.8%) felt isolated by their family members and friends because of their hospital work, while a low percentage (4.3%) indented to take a leave to avoid infection. However, worry and degree of worry were significantly associated with intended absenteeism (p < 0.0005), restriction of social contacts (p < 0.0005), and psychological distress (p = 0.036). Perceived sufficiency of information about several aspects of the A/H1N1 influenza was moderately high, and the overall information about the A/H1N1 influenza was considered clear (median 7.4/9). Also, perceived sufficiency of information for the prognosis of the infection was significantly independently associated with the degree of worry about the pandemic (p = 0.008).

**Conclusions:**

A significant proportion of HCWs experienced moderately high anxiety about the pandemic, and their degree of worry was an independent correlate of psychological distress. Since perceived sufficiency of information about the A/H1N1 influenza prognosis was associated with reduced degree of worry, hospital managers and consultation-liaison psychiatry services should try to provide for HCWs' need for information, in order to offer favourable working conditions in times of extreme distress, such as the current and future pandemics.

## Background

In April 2009, a new influenza A/H1N1 virus infection (known also as swine flu) emerged in Mexico and soon spread around the world. In June 2009, the World Health Organization (WHO) declared a phase 6 pandemic (the first pandemic in the 21^st ^century and the first in 41 years), when sustained community spread of swine flu occurred in multiple WHO regions [1]. By August 2009, there had been over 209,438 cases of influenza A/H1N1 worldwide, and at least 2,185 deaths had been reported to WHO [2]. In Greece, after a moderate wave during July and August 2009, in which 1,839 cases of A/H1N1 influenza had been confirmed and 1 death had been reported [3], a new wave started in the middle of September, which peaked in the middle of November, with the number of confirmed cases in Greece increasing to 13.899, with 59 deaths by the end of the December, 2009 [4].

Although A/H1N1 influenza pandemic, as opposed to nosocomial infections like the Severe Acute Respiratory Syndrome (SARS), is a community spread infection for which the risk of contagion is fairly evenly distributed across the population, public health efforts to contain the spread of the A/H1N1 influenza resulted in infection control measures that dominated hospital procedures and policies. Thus, health care workers (HCWs) assumed a key role, encountering an increasing workload and a perception of increased risk of infection, as in any infectious disease outbreak, and this could possibly affect their psychological well-being.

Research on the impact that previous disease outbreaks had on the psychological well-being of HCWs has shown that many HCWs presented high levels of psychological distress, frequent concerns regarding their health and their families' health, worries about their functional ability and fears of stigmatization [5-10]. HCWs worries and psychological distress over the previous SARS outbreak have been associated with higher job stress, social isolation and health fears [5,6]. Also, among the factors that have been found to be associated with HCWs' psychological distress in previous infectious disease outbreaks were physical and emotional exhaustion because of an overburdened healthcare system [11], rapidly changing medical information and procedures [5,12], media scrutiny [5,12], being a nurse [13], perception of risk to themselves [6], lifestyle affected by the disease outbreak [13], and personal vulnerability [8,9]. It has been also reported that when facing a possibility of an influenza pandemic, a remarkable proportion of HCWs did not go to work, despite a strong sense of duty [14-17].

Identifying factors in the health care environment that may be associated with HCW worries and psychological distress regarding infectious disease outbreaks and understanding their role in motivating HCWs to engage or to avoid their duties may help to provide HCWs with the most favourable working conditions possible in times of extreme distress [6]. No studies, however, have investigated HCWs' worries, concerns or psychological distress at the height of the epidemic of the new strain of influenza virus, A/H1N1. Prompted by this fact, the aim of the present study was to assess HCWs' acute responses to A/H1N1 influenza pandemic as indicated by their degree of concerns and worries over the pandemic, their degree of perceived sufficiency of information concerning A/H1N1 influenza, their intended behavior during the pandemic and whether these factors were associated with psychological distress.

## Methods

The study was carried out between September 1^st ^and 30^th^, 2009, at the University General Hospital of Ioannina, Greece, a tertiary teaching hospital with 850 beds providing secondary and tertiary care for a general population of 350,000. After the first moderate wave of the A/H1N1 influenza pandemic in July and August, 2009, there was in the beginning of September an increasing public concern and fears about a new pandemic outbreak in autumn. Indeed, during the study, 8.8% of the referred patients were confirmed A/H1N1 influenza cases, a proportion which increased rapidly, reaching a peak in the middle of November with 65.8% confirmed A/H1N1 influenza cases, which decreased to 46.7% by the end of December, 2009. Overall, from September, 2009, to the end of December, 2009, 838 confirmed A/H1N1 influenza cases were referred to our hospital, without any reported deaths.

To assess HCWs' concerns and worries over the pandemic, their degree of perceived sufficiency of information concerning A/H1N1 influenza, their intended behavior during the pandemic and whether these factors were associated with psychological distress, the following self-reported questionnaires were administered, along with a request for demographic characteristics:

(1) A questionnaire developed for this study comprising 20 items, which were chosen based on the available literature on the perceptions and opinions of experts regarding infectious disease outbreaks [Additional files 1 &2]. Eight items were dichotomous (Y/N) and 12 items were scored on a 9-point Likert scale from *very little *(1) to *very much *(9), *very low *(1) to *very high *(9) or *strongly agree *(9) to *strongly disagree *(1) (Tables 1, 2 &3). Items were grouped in seven domains: (a) HCWs' concerns, worries and degree of worry about the A/H1N1 influenza pandemic, (b) perceived sufficiency of information, (c) confidence in information received and measures taken by their department regarding A/H1N1 influenza pandemic, (d) perception of personal risk, (e) perception of being a risk to others (e.g. family and friends), (f) perception of stigmatization, isolation and sense of duty, and (g) work satisfaction. Cronbach's α score for factors consisting of items related to perceived information and information needs ranged from 0.68 to 0.89. Part-I of Cassileth's Information Styles Questionnaire [18] was also embedded in the questionnaire, which assesses the amount of desirable informational details about health issues required by the hospital worker, on a 5-point Likert scale.

**Table 1:** Healthcare workers' concerns and worries about A/H1N1 influenza pandemic.

	Total (N = 469)	1. Nurse (N = 209)	2. Medical (N = 120)	3. Allied (N = 59)	4. Auxiliary (N = 81)	p-value
I worry about the A/H1N1 influenza pandemic (Y/N; *N*, %)	266 (56.7)	127 (60.7)	43 (35.8)	33 (55.9)	53 (65.4)	< 0.0005 ^(a)^

Degree of worry (mean ± SD; median) (1: I have very little worry; 9: I'm very much worried)						
In the entire sample	4.2 ± 2.2; 4/9	4.4 ± 2.2	3.3 ± 1.8 ^1 (c)^	4.0 ± 2.2	5.3 ± 2.3 ^1,2,3^	< 0.0005 ^(b)^
In those who answered they worried	5.6 ± 1.7; 6/9	5.5 ± 1.6	5.0 ± 1.2	5.4 ± 1.7	6.2 ± 1.9 ^2^	0.005 ^(b)^

I mostly worry about: (Y/N; *N*, %)						
The disease's danger	146 (54.9)	76 (59.8)	19 (44.2)	16 (48.5)	30 (56.6)	< 0.0005 ^(a)^
The risk for family and relatives to be infected	161 (60.5)	84 (66.1)	27 (62.8)	22 (66.7)	24 (45.3)	0.015 ^(a)^
Isolation from family and/or social environment	34 (12.8)	18 (14.2)	4 (9.3)	4 (12.1)	7 (13.2)	0.294 ^(a)^
The consequences on my functional ability	115 (43.2)	59 (46.4)	16 (37.2)	14 (42.4)	24 (45.3)	0.007 ^(a)^

Perceived risk for being infected by the A/H1N1 virus (1: very low, 9: very high; mean ± SD; median)	6.2 ± 2.3; 6/9	6.4 ± 2.1	6.2 ± 2.0	5.7 ± 2.1	6.2 ± 2.0	0.117 ^(b)^
I think that being infected with the A/H1N1 influenza would have major consequences on my health (1:strongly disagree, 9: strongly agree; mean ± SD; median)	4.5 ± 2.2; 5/9	4.7 ± 2.2 ^2,4^	3.4 ± 1.8 ^1,3,4^	4.3 ± 2.3 ^2,4^	5.6 ± 2.0 ^1,2,3^	< 0.0005 ^(b)^
I believe that the infection is difficult to treat (1:strongly disagree, 9: strongly agree; mean ± SD)	4.8 ± 2.0; 5/9	4.9 ± 2.0	4.2 ± 1.9 ^1,4^	4.6 ± 2.1	5.2 ± 1.7	0.002 ^(b)^
I feel that my department is well prepared for the A/H1N1 influenza pandemic (1:strongly disagree, 9: strongly agree; mean ± SD; median)	4.7 ± 2.4; 5/9	4.2 ± 2.3 ^2,3,4^	5.0 ± 2.4	5.2 ± 2.0	5.1 ± 2.7	0.003 ^(b)^
I think it would be important if there was a service offering psychological support regarding my concerns about the pandemic (1:strongly disagree, 9: strongly agree; mean ± SD; median)	5.6 ± 2.9; 6/9	5.9 ± 2.9	4.5 ± 2.7 ^1,3,4^	6.5 ± 2.3	5.9 ± 3.1	< 0.0005 ^(b)^

(a) chi-square test; (b), ANOVA; (c) Significant differences between this category and the categories mentioned by numbers (Bonferroni post-hoc tests)

**Table 2:** Healthcare workers' perceived sufficiency of information about A/H1N1 influenza pandemic and general health information needs (mean ± SD).

	**Total (N = 469) (mean ± SD; median)**	**1. Nurse (N = 209)**	**2. Medical (N = 120)**^**(b)**^	**3. Allied (N = 59)**	**4. Auxiliary (N = 81)**	**p-value**^**a**^
	
I believe that I have heard sufficient information about: (1:strongly disagree, 9: strongly agree)						
A/H1N1 influenza symptoms	7.2 ± 1.8, 8/9	7.2 ± 1.8	7.8 ± 1.6 ^1,3,4^	7.0 ± 1.7	6.6 ± 1.8	< 0.0005
A/H1N1 influenza prognosis	6.4 ± 2.1, 7/9	6.1 ± 2.1	7.1 ± 1.9 ^1,3,4^	6.1 ± 2.1	6.0 ± 2.1	< 0.0005
A/H1N1 influenza treatment	6.5 ± 2.1, 7/9	6.3 ± 2.1	7.3 ± 1.8 ^1,3,4^	6.2 ± 2.0	5.9 ± 2.3	< 0.0005
A/H1N1 influenza infection route	7.5 ± 1.8, 8/9	7.3 ± 1.7	8.1 ± 1.5 ^1,3,4^	7.3 ± 1.8	7.1 ± 1.9	< 0.0005
A/H1N1 influenza preventive measures	7.2 ± 2.0, 8/9	6.9 ± 2.1	7.8 ± 1.7 ^1,3,4^	7.1 ± 2.0	6.9 ± 2.1	< 0.0005
I believe that my department provided clear information about the A/H1N1 influenza pandemic (1:strongly disagree, 9: strongly agree)	5.4 ± 2.4, 6/9	5.4 ± 2.4	5.3 ± 2.5	5.5 ± 2.2	5.9 ± 2.8	0.408
Overall, the information I have heard about the A/H1N1 influenza has been clear (1:strongly disagree, 9: strongly agree; five items, Cronbach's α, 0.89)	7.0 ± 1.6, 7.4/9	6.8 ± 1.7	7.6 ± 1.4 ^1,3,4^	6.7 ± 1.5	6.5 ± 1.7	< 0.0005
General health-information needs (1: for a disease that I might suffer, I prefer having no more information than needed; 5: I prefer as much information as possible)	3.6 ± 1.6, 4/5	3.8 ± 1.5	3.3 ± 1.6	3.2 ± 1.5	3.5 ± 1.6	0.036

(a), One-way ANOVA; (b) Significant differences between this category and the categories mentioned by numbers (Bonferroni post-hoc tests)

**Table 3:** Intended behavior associated with worry and the degree of worry about the A/H1N1 influenza pandemic.

		Do you worry about the new A/H1N1 influenza pandemic?			Degree of worry		
			
		Yes (N = 266)	No (N = 203)	p-value	mean ± SD	p-value	***d ***^***4***^
Restriction of Social Contacts (I have restricted my social contacts because my work environment is considered "dangerous")	Yes	27 (10.2%)	4 (2.0%)	< 0.0005^1^	6.1 ± 1.9	< 0.0005^2^	0.917
	No	239 (89.8%)	199 (98.0%)		4.1 ± 2.2		

Isolation (I feel that my family members and friends avoid contacts with me, because I work in a "high-risk" environment)	Yes	14 (5.3%)	4 (2.0%)	0.05^1^	5.6 ± 2.2	0.016^2^	0.636
	No	252 (94.7%)	199 (98.0%)		4.2 ± 2.2		

Intended work avoidance (Lately I have been so concerned about the A/H1N1 influenza that I would take a leave to avoid going to work)	Yes	18 (6.8%)	2 (1.0%)	0.003^1^	6.1 ± 2.3	< 0.0005^2^	0.952
	No	248 (93.2%)	201 (99.0%)		4.0 ± 2.2		

Sense of Duty (mean ± SD) (In an emergency situation due to the A/H1N1 influenza pandemic, how possible would it be to avoid your duties?) (1 = highly possible, 9 = not at all possible)		5.4 ± 2.8	6.4 ± 2.7	< 0.0005^2^	-0.156^3^	0.001	0.315

1, chi-square tests; 2, two-tailed t-tests; 3, Pearson correlation; 4, Cohen's *d *as effect size

(2) To assess HCWs psychological distress, the General Health Questionnaire-28 (GHQ-28) [19] was used. It comprises 28 items and, according to its standardization for the Greek population [20], scores above 5 are indicative of mild to moderate psychological distress. Studies have also shown that scores above 11 are indicative of severe psychological distress [21]. As the total GHQ-28 score exceeds this recommended cut-off point, the probability of being assessed as having a psychiatric diagnosis at interview increases.

### Recruitment

HCWs in all the hospital's departments and clinical units, including the intensive care unit and the emergency department, were asked to participate in the study. A letter was sent to each department director, informing them about the study and asking for their permission to distribute the questionnaires to employees in their departments. All contacted departments agreed to participate. In a scheduled meeting with each department's staff, an estimated number of 1000 potential participants were informed about the aims of the study and that participation was voluntary. Blank questionnaires with sealed boxes for collection of responses were placed at various designated work areas in each participating unit. Four hundred sixty-nine subjects (response rate: 46.9%) returned the survey after an informed consent was obtained. The participants were divided into four groups: Medical staff, nursing staff, allied personnel (physiotherapists, social workers, psychologists, etc) and auxiliary staff (domestic services, administration, etc). All the procedures followed were in accordance with the ethical standards on human experimentation (World Medical Association Helsinki Declaration) and were approved by the Ioannina University General Hospital's responsible ethics committee (38/31.8.2009).

### Statistical analysis

All the statistical analyses were performed using the Statistical Package for the Social Sciences (SPSS) 15.0 (SPSS Inc., Chicago, IL, USA) for Windows. Summary statistics for all variables were calculated. Normality was tested by the Kolmogorov-Smirnov test [22]. Chi-square analyses and one-way Analyses of Variance (ANOVA) were performed to assess differences in concerns, worries, degree of worry and perceived sufficiency of information among the groups of the hospital staff. To assess the relationship of worries and degree of worry with intended behaviour, chi-square analyses for categorical data (e.g. worries [Y/N], or intended behaviour [Y/N]) and two-tailed t-tests for continuous data (e.g. degree of worry) were carried out [22]. Univariate comparisons were conducted next to assess variables associated with the degree of worry about the A/H1N1 influenza pandemic and the HCWs' psychological distress. Chi-square analyses for categorical data (e.g. for testing the relationship of "psychiatric caseness" [GHQ < 5/GHQ > 5] with gender [M/F]), two-tailed t-tests for continuous data (e.g. for testing the relationship of degree of worry with gender [M/F]) and Pearson correlations for testing the relationship between continuous variables (e.g. between degree of worry and age) were carried out [22]. To assess the factors most closely associated with HCWs' degree of worry, a multiple regression analysis was carried out with dependent variable the degree of worry and independent variables the major demographic variables and the statistically significant variables of the previous univariate analyses. Missing data of independent variables were treated by mean substitution. Colinearity between independent variables was tested based on variance inflation factors (VIF) and tolerances for individual variables [23]. Finally, a binary logistic regression analysis was used to identify the multivariate associations of HCW's psychological distress adjusting for demographic variables. The dichotomous dependent variable was "total GHQ score" and the cut-off point was "5", as found by receiver-operating characteristics analysis for the GHQ-28 in the Greek population [20]. Selection of independent variables was based on the results of the previous univariate analyses.

## Results

### Demographic characteristics and psychological distress

Table 4 presents the participants' major demographic and professional characteristics and their scores on psychological distress measures. Ninety-seven out of the 469 participants (20.7%) presented scores on GHQ-28 > 5, indicative of mild to moderate psychological distress, with a higher proportion of nurses (23.9%) and medical staff (21.7%) presenting scores >5, as compared to allied (13.5%) and auxiliary (12.3%) personnel (p = 0.030). Of the 97 participants who presented scores >5, 32 (6.8% of the entire sample) presented scores >11, indicative of severe psychological distress. Again, the number of nurses (8.6%) and medical staff (6.7%) who presented "severe psychological distress" was also higher, in comparison to allied (1.7%) and auxiliary (3.7%) personnel (p = 0.033).

**Table 4:** Sample characteristics and psychological distress (N = 469)

Demographics	
Age (years) [mean ± SD]	38.4 ± 8.7
Female Gender, N (%)	321 (68.4%)
Living alone, N (%)	147 (31.3%)
Education, N (%)	
Below primary	13 (2.8%)
Primary	14 (3.0%)
High-School	159 (33.9%)
College/University	283 (60.3%)
Children; yes, N (%)	296 (63.1%)
Profession, N (%)	
Nurse	209 (44.6%)
Medical	120 (25.6%)
Allied	59 (12.6%)
Auxiliary	81 (17.3%)
Mild to Moderate Psychological Distress (GHQ-28 > 5), N (%)	97 (20.7%)
Severe Psychological Distress (GHQ-28 > 11), N (%)	32 (6.8%)

### Concerns and worries about the A/H1N1 influenza pandemic

As shown in Table 1, 56.7% of the participants reported they were worried about the A/H1N1 influenza pandemic, their scores of their degree of worry being indicative of moderately high concern (median, 6/9). The proportion of nurses, auxiliary staff, and allied personnel that reported that they were worried was higher compared to that of the medical staff (p < 0.0005). Auxiliary staff presented higher degree of worry than all other groups, and nurses were also more worried than medical staff (p < 0.0005). The most frequent concern was the risk of infection of family and friends, followed by worries about the dangerousness of the disease and its consequences on their functional ability. Significantly more nurses and auxiliary staff worried about the danger of the disease (p < 0.0005). Concerns about isolation from family and/or social environment were low across all groups (9.3% to 14.2%).

The perceived risk of being infected by the A/H1N1 virus was considered moderately high (median, 6/9), with no significant differences among groups. Over 50% of the participants agreed to some extent that being infected with the A/H1N1 influenza would have a major consequence on their health (median, 5/9), with medical staff presenting the lower rates and auxiliary staff presenting the higher rates (p < 0.0005). More than half also agreed to some degree that the infection is difficult to treat (median, 5/9), with medical staff again presenting the lower rates.

More than half the HCWs agreed to some degree that the ward/department where they worked was well prepared for the A/H1N1 influenza pandemic (median, 5/9), with nurses presenting the lower rates of agreement in this statement, compared to the opinion of the other professionals. Finally, the existence of a service offering psychological support regarding the HWCs' concerns about the A/H1N1 influenza was considered of moderately high importance (median, 6/9), and medical staff were less likely to perceive this need.

### Perceived sufficiency of information about the H1N1 influenza pandemic

As shown in Table 2, the degree of HCWs' perceived sufficiency of information about the A/H1N1 influenza symptoms, prognosis, treatment, contagion route, and preventive measures could be considered moderately high to high (medians ranged from 7/9 to 8/9). A significant proportion of HCWs agreed to some degree that the information provided by the workers' department was clear (median, 6/9), while the overall information about the A/H1N1 influenza was considered clear, as well (median, 7.4/9). Medical staff reported the highest rates of perceived sufficiency of information, compared to the other groups.

Regarding general health information needs, a significant proportion of the staff (43.7%) reported that for a disease that they might suffer, they preferred as much information as possible. An additional 36.3% wanted "a few more", "some more" or "many more" informational details, while one fifth (20.0%) wanted only to know the information needed to care for themselves properly and wished to avoid additional details. The overall mean and median for general information needs are presented in Table 2. There were minimal differences among the staff categories, with nurses preferring more frequently as much information as possible (50.0%, compared to 41.5% of the medical staff and to 37.3% of the allied and auxiliary personnel).

### Worry and intended behavior

As shown in Table 3, few HCWs (N = 31, 6.6%) reported that they had restricted their social contacts because they considered their work environment "dangerous" and 18 (3.8%) felt that family members and friends avoided them because of their hospital work. Along the same lines, few HCWs (N = 20, 4.3%) reported that they would take a leave to avoid infection. However, worries and degree of worry were significantly associated with intended behaviors, mainly with restriction in social contacts and intentional absenteeism.

### Factors associated with the degree of worry about the *A/H1N1 *influenza pandemic

Univariate analyses revealed a number of variables associated with the degree of worry about the A/H1N1 influenza pandemic (Table 5). Subsequent multiple regression analysis showed that perceived information about the A/H1N1 influenza prognosis was the variable most closely negatively associated with the degree of worry (p = 0.008). On the other hand, being auxiliary staff (p = 0.023), believing that being infected with the A/H1N1 influenza would have major health consequences (p < 0.0005) and that the infection would be difficult to treat (p = 0.003) were the variables most closely positively associated with the degree of worry.

**Table 5:** Variables associated with the hospital staffs degree of worry about A/H1N1 influenza pandemic (N = 469).

**Independent Variables**	**Univariate Analyses**	**Multiple Regression Analysis **^**(1)**^	
		
	**p-values**	**beta ^(2)^**	**p-values**
	
Demographics			
Sex	0.201 ^(3)^	0.053	0.213
Age	0.006 ^(4)^	0.024	0.620
Educational Level	< 0.0005 ^(4)^	0.040	0.396
Children (No = 0, Yes = 1)	0.003 ^(3)^	0.047	0.338
Profession			
Nurse	0.129 ^(3)^	-	-
Medical	< 0.0005 ^(3)^	-0.070	0.140
Allied	0.547 ^(3)^	-	-
Auxiliary	< 0.0005 ^(3)^	0.099	0.023
Perceived sufficiency of information about:			
A/H1N1 influenza symptoms	0.006 ^(4)^	-0.086	0.175
A/H1N1 influenza prognosis	< 0.0005 ^(4)^	-0.161	0.008
A/H1N1 influenza treatment	< 0.0005 ^(4)^	-0.005	0.993
A/H1N1 influenza infection route	0.017 ^(4)^	-0.019	0.781
A/H1N1 influenza preventive measures	0.004 ^(4)^	-0.002	0.995
Beliefs about a possible infection			
It would have major health consequences	< 0.0005 ^(4)^	0.368	< 0.0005
It would be difficult to treat	< 0.0005 ^(4)^	0.154	0.003
Department's efficacy			
They felt the department had provided clear information about the A/H1N1 influenza	0.510 ^(4)^	-	-
They felt their department was well prepared for the A/H1N1 influenza pandemic	0.637 ^(4)^	-	-
Work satisfaction	0.298 ^(4)^	-	-

(1): Multiple regression analysis with dependent variable the degree of worry about the swine flu pandemic and independent variables the major demographic variables and the statistically significant variables of the univariate comparisons; Cumulative R^2 ^Adjusted= 0.270; F_[13,455] _= 14.3, p < 0.0005 (2): Standardized beta coefficients; (3): two-tailed t-test; (4): Pearson correlation; All the VIFs for individual variables were less than 2 and all tolerances were close to 1.

### Factors associated with hospital staff's psychological distress

As shown in Table 6, univariate analyses revealed a number of variables associated with general psychological distress. Subsequent multiple logistic regression analysis showed that the degree of worry about the A/H1N1 influenza pandemic was significantly independently associated with psychological distress (p = 0.036). Additionally, the odds of being assessed with moderate or severe psychological distress upon interview were 2.2 times greater among nurses and 4.5 times greater among auxiliary staff, compared to medical and allied staff, while work satisfaction was independently negatively associated with psychological distress.

**Table 6:** Variables associated with the hospital staff's general psychological distress (N = 469).

**Independent Variables**	**Univariate Analyses**	**Multivariate Logistic Regression Analysis **^**(1)**^	
		
	**p-values**	**Odds ratios (95% CI) **^**(2) **^	**p-values**
	
Demographics			
Sex	0.052 ^(3)^	1. 1 (0.59 - 2.07)	0.751
Age	0.559 ^(4)^	1. 0 (0.96 - 1.03)	0.994
Educational Level	0.257 ^(4)^	0. 8 (0.66 - 1.09)	0.201
Living alone	0.268 ^(3)^	-	-
Profession			
Nurse	0.019 ^(3)^	2. 2 (0.59 - 2.07)	0.046
Medical	0.766 ^(3)^	-	-
Allied	0.037 ^(3)^	1. 7 (0.64 - 4.71)	0.274
Auxiliary	0.024 ^(3)^	4. 5 (1.38 - 14.70)	0.013
Degree of worry about swine flu pandemic	0.005 ^(4)^	1. 2 (1.01 - 1.35)	0.036
Perceived sufficiency of information about:			
A/H1N1 influenza symptoms	0.019 ^(4)^	0. 9 (0.81 - 1.17)	0.760
A/H1N1 influenza prognosis	0.789 ^(4)^	-	-
A/H1N1 influenza treatment	0.026 ^(4)^	0. 9 (0.80 - 1.13)	0.581
A/H1N1 influenza infection route	0.335 ^(4)^	-	-
A/H1N1 influenza preventive measures	0.131 ^(4)^	-	-
Beliefs about a possible infection			
It would have major health consequences	0.044 ^(4)^	1. 0 (0.86 - 1.23)	0.739
It would be difficult to treat	0.001 ^(4)^	1. 0 (0.91 - 1.29)	0.376
Department's efficacy			
They felt the department had provided clear information about the A/H1N1 influenza	0.049 ^(4)^	1. 0 (0.88 - 1.17)	0.838
They felt their department was well prepared for the A/H1N1 influenza pandemic	0.001 ^(4)^	0. 9 (0.76 - 1.01)	0.060
Work satisfaction	< 0.0005 ^(4)^	0. 8 (0.73 - 0.95)	0.009

(1): Multivariate logistic regression analysis with dependent variable the General Health Index of GHQ-28 and independent variables the major demographic variables and the statistically significant variables of the univariate comparisons. The predictive values were calculated based on the probability of being "psychiatric case" and the cut-off value between "case" and "non-case" was 0.500. The multivariate regression analysis correctly classified 78.6% of the cases, with a Nagelkerke R Square = 0.147; (2): 95% confidence interval; (3): chi-square tests; (4): two-tailed t-test; All the VIFs for individual variables were less than 2 and all tolerances were close to 1.

## Discussion

The results of the present study showed that in September, 2009, when there were significant public and hospital staff concerns about a new A/H1N1 influenza pandemic outbreak, more than half of our hospital's HCWs (56.7%) reported they worried about the pandemic, their degree of worry being moderately high. The most frequent concern was for infection of family and friends and the consequences of the disease on their health. The perceived risk for being infected was considered moderately high and more than half agreed to some degree that being infected with the A/H1N1 influenza would have a major consequence on their health. Few HCWs (6.6%) had restricted their social contacts and fewer (3.8%) felt isolated by their family members and friends because of their hospital work, while a low percentage (4.3%) would take a leave to avoid infection. However, worries and degree of worry were significantly associated with intentional absenteeism, restriction of social contacts, and psychological distress. Perceived sufficiency of information about several aspects of the A/H1N1 influenza was moderately high, and the overall information about the A/H1N1 influenza was considered clear. Although more than half agreed to some degree that the ward/department they worked was well prepared for the pandemic, a significant proportion (44.4%) expressed a disagreement (at least somewhat) in this respect, with nurses presenting the lower rates of agreement. Finally, perceived sufficiency of information for the prognosis of the infection was the variable most closely independently associated with the degree of worry about the pandemic.

It has been well established that HCWs experienced significant stress during infectious epidemics [5-9]. Reports of the psychological impact of SARS on hospital staff indicated that high levels of distress were common [5]. In Singapore, over 27% of HCWs had a GHQ-28 score >5 and approximately 20% of the doctors and nurses were suffering from PTSD [24]. In one Toronto hospital, 29% of respondents scored above the threshold for "emotional distress" on GHQ-12 [17], while in Taiwan, 5% of staff members suffered from an acute stress disorder [7]. Studies on HCWs' concerns about the avian influenza pandemic have also shown that 71.6% had significant concerns and had perceived the pandemic as having adverse impacts on their personal life and work [10]. Despite, however, the large epidemiological literature on the A/H1N1 influenza pandemic, there is little information available regarding the worries, concerns or the psychological impact that the pandemic might have on HCWs. To the best of our knowledge, at the time of reporting, this is the first study investigating the acute concerns and worries of hospital staff about the A/H1N1 influenza pandemic and their associations with psychological distress.

Our results showed that more than half of HCWs experienced moderately high levels of worry about the pandemic, with auxiliary staff being more worried than all other groups and nurses being more worried than medical staff. On the other hand, our findings also showed that 20.7% of HCWs presented scores indicating mild to moderate psychological distress, rates similar to those found in a previous study, at times when there was no infectious exposure, when 18.1% of HCWs presented scores indicating mild to moderate psychological distress [25]. In another study of 275 HCWs in the same hospital one year before the infectious outbreak, we found that 21.8% presented scores indicative of mild or moderate psychological distress (unpublished data). These findings indicate that psychological distress is a common experience in HCWs, as also has been suggested by studies in the UK, which have shown that among doctors and nurses, between 28 and 32% scored above the threshold for "emotional distress" in GHQ-12 [26-28]. However, despite the fact that HCWs' psychological distress was not elevated in the present study, their degree of worry about the pandemic was an independent correlate of psychological distress (Table 6), indicating that HCWs concerns about the pandemic might contribute to psychological distress. The cross-sectional design of our study prevent us from answering questions about causality, since it is plausible, for instance, that people who are already distressed for reasons not measured in this study are more likely to worry about A/H1N1 influenza pandemic. Work satisfaction was also associated with psychological distress (Table 6) but it was not correlated to the degree of worry about the pandemic (Table 5), indicating that the association of work dissatisfaction with psychological distress could be attributed to the chronic stress and burnout that are common in hospital settings and leading contributors to work dissatisfaction [29] rather than the HCW's concerns about the pandemic.

Consistent with the results of previous studies in SARS-affected hospitals [17,30,31], nurses and medical staff presented high rates of psychological distress. Although by definition, both medical staff and nurses have greater contact with patients, medical staff expressed the lower degree of worry (Table 1), possibly because they mostly regarded themselves as sufficiently informed (Table 2). A greater proportion of nurses and auxiliary staff also worried about the A/H1N1 influenza pandemic compared to medical staff, and their degree of worry was also greater than that of doctors (Table 1). Auxiliary staff expressed the highest level of worry, while being a nurse and/or auxiliary staff was significantly associated with psychological distress. In addition, auxiliary staff considered the consequences of the infection for their health to be greater (Table 1). These findings indicate that assessing and intervening for the psychological impact of the infectious outbreak on HCWs, especially on nurses and auxiliary staff, is of particular importance in planning for the current and future outbreaks of infectious diseases. Hospital policies should also take into account auxiliary staff's concerns and worries, and since we found that perceived sufficiency of information was associated with lower degree of worry, hospital managers should try to provide for auxiliary staff's information needs, in order to provide a favourable working environment in times of extreme public health-related concerns, such as the current A/H1N1 influenza pandemic.

Nurses constitute the largest hospital occupational group and are directly and intensively involved in patient care, experiencing a greater risk of contagion in cases of infectious diseases. It is therefore not surprising that nurses reported the higher percentages when asked whether, for a disease that they might contract, they would prefer as much information as possible, while they also felt their department was less well prepared for the A/H1N1 influenza pandemic, compared to the other staff groups (Table 1). Other studies have also found that in a pandemic of influenza, medical and nursing staff were significantly less likely than ancillary and support staff to consider their ward/department sufficiently prepared for the pandemic [32]. Our results and the results of the aforementioned studies indicate that hospital and department managers and directors should consider the opinions of nurses and medical staff with respect to the proper ward/department preparation for a pandemic, if they are to offer the most favourable working conditions possible for HCWs in times of extreme distress, such as the current and future infection pandemics.

Consistent with the results of other studies reporting that distress can be amplified in the face of lack of clear information that is common in the initial period of disease outbreaks [33], in our study perceived sufficiency of information about the A/H1N1 influenza prognosis was independently associated with reduced degree of worry (Table 5).

HCWs across a range of professions tended to feel motivated to work during the A/H1N1 influenza pandemic, as indicated by the high sense of duty expressed and by the low proportion of HCWs reporting that they would take a leave to avoid infection (4.3%). In addition, although fear of stigmatization, in the form of being avoided by family and friends, was observed to be a prominent aspect of many HCWs' experience during SARS [5,7,16,17], this was not the case in the present study, where only 3.8% felt that family and friends avoided them because of their hospital work, and only 6.6% had restricted their social contacts themselves. This is not surprising, since the stigmatization reactions of the public to SARS was founded on the fact that the infection was limited to hospitals, whereas the A/H1N1 infection is a community spread infection for which the risk of infection is fairly evenly distributed across the population. Nevertheless, studies in other countries have shown variable rates of intentional absenteeism due to contagious diseases [12-14,32], depending on the kind of the infection, the different time periods the studies took place, the different survey questions, and the cultural differences or religious beliefs. Our results indicated that most HCWs surveyed considered that it was not possible to avoid their duties in an emergency situation due to the pandemic and they would continue working despite the potential risks (Table 3), a finding consistent with the view that HCWs consider it unethical to abandon their professional responsibilities in order to protect themselves or their families [32,34,35].

The main methodological limitation of the present study lies in the response rate. An accurate estimation of the response rate was difficult, because the number of staff who could have been contacted during the 4-week period the study took place is difficult to estimate, given that in September many Greek employees are on vacation. However, the response rate of this study (46.9%) is comparable to other studies investigating HCWs' concerns and distress about infectious outbreaks (for example, 47% in the study of Nickell et al [17], 41.1% in the study of Styra et al [6], or 23.3% in the study of Maunder et al [5]). In addition, comparison of the characteristics of the study sample with the total hospital employees suggested that the study sample is representative of HCWs in our hospital. For example, nurses comprise 46.4% of the hospital staff and 44.6% of the study sample. Despite this, however, we cannot refute the criticism that an underlying response style might have led to our results. We also cannot exclude the possibility that particularly concerned or distressed HCWs were under-represented, as such subjects might be on leave because of their worries about the pandemic and thus unable to join the study. Further methodological limitations are the potential for bias caused by socially acceptable answering resulting in possible underestimated intentional absenteeism rates. Gathering information regarding psychological distress by self-report is a further limitation. Finally, as mentioned earlier, questions about causality cannot be resolved by cross-sectional surveys, and therefore future prospective studies are needed to confirm our findings and to investigate the causal paths of the associations reported regarding HCWs concerns and worries about A/H1N1 influenza pandemic, psychological distress, perceived information and intended behaviour.

## Conclusions

In conclusion, our study showed that a significant proportion of HCWs experienced moderately high levels of worry about the pandemic, and their degree of worry was an independent correlate of psychological distress. Few HCWs had restricted their social contacts, while a low percentage would take a leave to avoid infection. However, worries and degree of worry were significantly associated with intentional absenteeism and restriction of social contacts. Since perceived sufficiency of information about the A/H1N1 influenza was associated with reduced degree of worry, hospital policies should try to provide for HCWs' information needs, in order to provide the best possible working conditions in times of extreme public and hospital staff distress, such as the current A/H1N1 influenza pandemic.

## Study highlights

1) What is current knowledge:

• Health care workers (HCWs) presented frequent concerns regarding their health and their families' health, high levels of psychological distress, worries about their functional ability and fears of stigmatization during previous disease outbreaks.

• HCWs' worries and psychological distress over the previous SARS outbreak have also been associated with higher job stress, social isolation and intentional absenteeism.

• No studies have investigated HCWs' worries, concerns or psychological distress during the peak of the new strain of influenza virus, A/H1N1 and it is not known whether the previous SARS experience is representative or unique.

2) What is new here:

• In September, 2009, during the A/H1N1 pandemic, more than half of HCWs experienced moderately high levels of worry about the pandemic, with auxiliary staff presenting the higher degree of worry and nurses being more worried than medical staff.

• The most frequent concern was for infection of family and friends and the consequences of the disease on their health.

• The degree of worry about the pandemic was an independent correlate of psychological distress.

• Few HCWs (6.6%) had restricted their social contacts and fewer (3.8%) felt isolated by their family members and friends because of their hospital work, while a low percentage (4.3%) would take a leave to avoid infection. However, worries and degree of worry were significantly associated with intentional absenteeism and restriction of social contacts.

• Most HCWs considered that it was not possible to avoid their duties in an emergency situation due to the pandemic and they would continue working despite the potential risks.

• Perceived sufficiency of information about several aspects of the A/H1N1 influenza was moderately high, while perceived sufficiency of information about the A/H1N1 influenza prognosis was independently associated with reduced degree of worry.

• Hospital managers and consultation-liaison psychiatry services should try to provide for HCWs' information needs, if we are to offer favourable working conditions in times of extreme distress, such as the current and future pandemics.

## Competing interests

The authors declare that they have no competing interests.

## Authors' contributions

PG: participated in the conception and experimental design of the study, data analysis and preparation of the manuscript

CM: participated in the conception and experimental design of the study, in the data collection and preparation of the manuscript

DD: participated in data collection and preparation of the manuscript

DM: participated in data collection and preparation of the manuscript

TH: Conception and experimental design of the study, co-ordinated the study, data analysis, planning and drafting the manuscript

All authors state they have read and approved the final draft submitted

## Pre-publication history

The pre-publication history for this paper can be accessed here:

http://www.biomedcentral.com/1471-2334/10/322/prepub

## Supplementary Material

Additional file 1 **Questionnaire for assessing hospital staff worries and perceived sufficiency of information during the A/H1N1 influenza pandemic (Greek version)**. This file contains the Greek battery administered to assess hospital staff worries, perceived sufficiency of information and associated psychological distress during the A/H1N1 influenza pandemic. Click here for file

Additional file 2 **Questionnaire for assessing hospital staff worries and perceived sufficiency of information during the A/H1N1 influenza pandemic (English version)**. This file contains the English translation of the questionnaire administered to assess hospital staff worries and perceived sufficiency of information during the A/H1N1 influenza pandemic. Click here for file

## References

[B1] WHO/Europe influenza surveillance http://www.euro.who.int/influenza/AH1N1/20091026_1

[B2] WHO/Global Alert and Response (GAR) - Pandemic (2009) Update 63 http://www.who.int/csr/don/2009_08_28/en/index.html

[B3] Hellenic Centre for Disease Control and Prevention (KEELPNO) http://www.keel.org.gr/keelpno/2009/id994/gripi_ebdo_20090826.pdf

[B4] Hellenic Centre for Disease Control and Prevention (KEELPNO) http://www.keel.org.gr/keelpno/2009/id994/gripi_ebdo_20091223.pdf

[B5] Maunder RG Lancee WJ Rourke S Hunter JJ Goldbloom D Balderson K Petryshen P Steinberg R Wasylenki D Koh D Fones C Factors associated with the psychological impact of severe acute respiratory syndrome on nurses and other hospital workers in Toronto Psychosomatic Medicine 2004 66 6 938 42 10.1097/01.psy.0000145673.84698.18 15564361

[B6] Styra R Hawryluck L Robinson S Kasapinovic S Fones C Gold WL Impact on health care workers employed in high-risk areas during the Toronto SARS outbreak Journal of Psychosomatic Research 2008 64 2 177 83 10.1016/j.jpsychores.2007.07.015 18222131 PMC7094601

[B7] Bai Y Lin CC Lin CY Chen JY Chue CM Chou P Survey of stress reactions among health care workers involved with the SARS outbreak Psychiatric Services 2004 55 9 1055 7 10.1176/appi.ps.55.9.1055 15345768

[B8] Tam CW Pang EP Lam LC Chiu HF Severe acute respiratory syndrome (SARS) in Hong Kong in 2003: stress and psychological impact among frontline healthcare workers Psychological Medicine 2004 34 7 1197 204 10.1017/S0033291704002247 15697046

[B9] Wong TW Yau JK Chan CL Kwong RS Ho SM Lau CC Lau FL Lit CH The psychological impact of severe acute respiratory syndrome outbreak on healthcare workers in emergency departments and how they cope European Journal of Emergency Medicine 2005 12 1 13 8 10.1097/00063110-200502000-00005 15674079

[B10] Cheong SK Wong TY Lee HY Fong YT Tan BY Koh GCh Chan KM Chia SE Koh D Concerns and preparedness for an avian influenza pandemic: a comparison between community hospital and tertiary hospital healthcare workers Industrial Health 2007 45 5 653 61 10.2486/indhealth.45.653 18057808

[B11] Koh D Lim MK Chia SE SARS: health care work can be hazardous to health Occupational Medicine (Oxford, England) 2003 53 4 241 3 12815118 10.1093/occmed/kqg090 PMC7107855

[B12] Maunder R The experience of the 2003 SARS outbreak as a traumatic stress among frontline healthcare workers in Toronto: lessons learned Philosophical Transactions of the Royal Society of London. Series B Biological Sciences 2004 359 1117 25 10.1098/rstb.2004.1483 PMC1693388 15306398

[B13] Nickell LA Crighton EJ Tracy CS Al-Enazy H Bolaji Y Hanjrah S Hussain A Makhlouf S Upshur RE Psychosocial effects of SARS on hospital staff: survey of a large tertiary care institution CMAJ 2004 170 793 8 14993174 10.1503/cmaj.1031077 PMC343853

[B14] Ives J Greenfield S Parry JM Draper H Gratus C Petts JI Sorell T Wilson S Healthcare workers' attitudes to working during pandemic influenza: a qualitative study BMC Public Health 2009 12 9 56 10.1186/1471-2458-9-56 PMC2654560 19216738

[B15] Damery S Wilson S Draper H Gratus C Greenfield S Ives J Parry J Petts J Sorell T Will the NHS continue to function in an influenza pandemic? A survey of healthcare workers in the West Midlands UK BMC Public Health 2009 14 9 142 10.1186/1471-2458-9-142 PMC2690584 19442272

[B16] Wicker S Rabenau HF Gottschalk R Influenza pandemic: Would healthcare workers come to work? An analysis of the ability and willingness to report to duty Bundesgesundheitsblatt Gesundheitsforschung Gesundheitsschutz 2009 52 8 862 9 10.1007/s00103-009-0913-6 19593535

[B17] Martinese F Keijzers G Grant S Lind J How would Australian hospital staff react to an avian influenza admission, or an influenza pandemic? Emergency Medicine Australasia 2009 21 1 12 24 10.1111/j.1742-6723.2008.01143.x 19254308 PMC7163727

[B18] Cassileth BR Zupkis RV Sutton-Smith K March V Information and participation preferences among cancer patients Annals of Internal Medicine 1980 92 832 836 7387025 10.7326/0003-4819-92-6-832

[B19] Goldberg DP Hillier VF A scaled version of the General Health Questionnaire Psychological Medicine 1979 9 139 45 10.1017/S0033291700021644 424481

[B20] Garyfallos G Karastergiou A Adamopoulou A Moutzoukis C Alagiozidou E Mala D Garyfallos A Greek version of the General Health Questionnaire: accuracy of translation and validity Acta Psychiatrica Scandinavica 1991 84 371 8 10.1111/j.1600-0447.1991.tb03162.x 1746290

[B21] Bridges KW Goldberg DP The validation of the GHQ-28 and the use of the MMSE in neurological in-patients British Journal of Psychiatry 1986 148 548 53 10.1192/bjp.148.5.548 3779225

[B22] Altman DG Practical statistics for medical research 1991 London: Chapman and Hall 285

[B23] Miles J Shevlin M Applying regression and correlation 2003 Sage, London 165 191

[B24] Chan AO Huak CY Psychological impact of the 2003 severe acute respiratory syndrome outbreak on health care workers in a medium size regional general hospital in Singapore Occupational Medicine (London) 2004 54 3 190 6 10.1093/occmed/kqh027 PMC7107861 15133143

[B25] Hyphantis TN Tsifetaki N Pappa C Voulgari PV Siafaka V Bai M Alamanos Y Drosos AA Mavreas V AA Drosos Clinical features and personality traits associated with psychological distress in systemic sclerosis patients Journal of Psychosomatic Research 2007 62 1 47 56 10.1016/j.jpsychores.2006.07.028 17188120

[B26] Ramirez AJ Graham J Richards MA Cull A Gregory WM Leaning MS Snashall DC Timothy AR Burnout and psychiatric disorder among cancer clinicians British Journal of Cancer 1995 71 1263 1269 7540037 10.1038/bjc.1995.244 PMC2033827

[B27] Firth-Cozens J Emotional distress in junior house officers BMJ 1987 295 533 536 10.1136/bmj.295.6597.533 3117213 PMC1247439

[B28] Hetherington A The Extent and Source of Stress in Emergency Care Report No. 9110 1993 UK, Cranfield University

[B29] Utriainen K Kyngäs H Hospital nurses' job satisfaction: a literature review Journal of Nursing Management 2009 17 8 1002 10 10.1111/j.1365-2834.2009.01028.x 19941574

[B30] Poutanen SM Low DE Henry B Finkelstein S Rose D Green K Tellier R Draker R Adachi D Ayers M Chan AK Skowronski DM Salit I Simor AE Slutsky AS Doyle PW Krajden M Petric M Brunham RC McGeer AJ National Microbiology Laboratory, Canada; Canadian Severe Acute Respiratory Syndrome Study Team for the National Microbiology Laboratory, Canada, and the Canadian Severe Acute Respiratory Syndrome Study Team Identification of severe acute respiratory syndrome in Canada New England Journal of Medicine 2003 348 1995 2005 10.1056/NEJMoa030634 12671061

[B31] Caputo KM Byrick R Chapman MG Orser BJ Orser BA Intubation of SARS patients: infection and perspectives of healthcare workers Canadian Journal of Anaesthesia 2006 53 122 9 10.1007/BF03021815 16434750

[B32] Seale H Leask J Po K MacIntyre CR "Will they just pack up and leave?" - attitudes and intended behaviour of hospital health care workers during an influenza pandemic BMC Health Services Research 2009 9 30 10.1186/1472-6963-9-30 19216792 PMC2661074

[B33] Johal SS Psychosocial impacts of quarantine during disease outbreaks and interventions that may help to relieve strain The New Zealand Medical Journal 2009 122 1296 47 52 19652680

[B34] Straus SE Wilson K Rambaldini G Rath D Lin Y Gold WL Kapral MK Severe acute respiratory syndrome and its impact on professionalism: qualitative study of physicians' behaviour during an emerging healthcare crisis BMJ 2004 329 7457 83 10.1136/bmj.38127.444838.63 15175231 PMC449811

[B35] Ehrenstein BP Hanses F Salzberger B Influenza pandemic and professional duty: family or patients first? A survey of hospital employees BMC Public Health 2006 6 311 10.1186/1471-2458-6-311 17192198 PMC1764890

