# Supplementary material for: General hospital staff worries, perceived sufficiency of information and associated psychological distress during the A/H1N1 influenza pandemic
Source: BMC Infect Dis. 2010 Nov 9;10:322. doi: 10.1186/1471-2334-10-322 (PMC2990753; doi:10.1186/1471-2334-10-322)
Supplement: Additional file 1 — Questionnaire for assessing hospital staff worries and perceived sufficiency of information during the A/H1N1 influenza pandemic (Greek version). This file contains the Greek battery administered to assess hospital staff worries, perceived sufficiency of information and associated psychological distress during the A/H1N1 influenza pandemic. [file 1471-2334-10-322-S1.DOC]

#
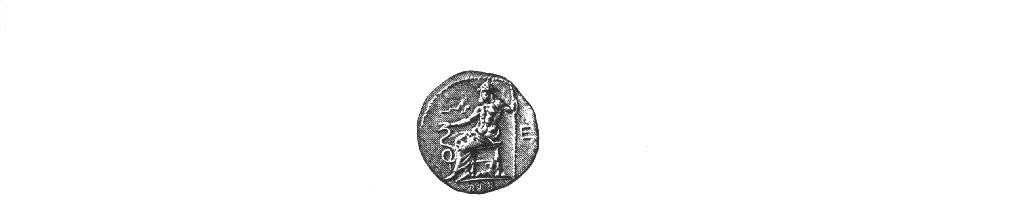
ΠΑΝΕΠΙΣΤΗΜΙΟ ΙΩΑΝΝΙΝΩΝ – ΙΑΤΡΙΚΗ ΣΧΟΛΗ

ΤΟΜΕΑΣ ΚΟΙΝΩΝΙΚΗΣ ΙΑΤΡΙΚΗΣ ΚΑΙ ΨΥΧΙΚΗΣ ΥΓΕΙΑΣ

**ΨΥΧΙΑΤΡΙΚΗ ΚΛΙΝΙΚΗ**

**ΔΙΕΥΘΥΝΤΗΣ: ΚΑΘΗΓΗΤΗΣ ΒΕΝΕΤΣΑΝΟΣ ΜΑΥΡΕΑΣ**

**ΜΟΝΑΔΑ ΣΥΜΒΟΥΛΕΥΤΙΚΗΣ-ΔΙΑΣΥΝΔΕΤΙΚΗΣ ΨΥΧΙΑΤΡΙΚΗΣ**

**ΕΠΙΣΤ. ΥΠΕΥΘΥΝΟΣ: ΘΩΜΑΣ ΥΦΑΝΤΗΣ, ΑΝΑΠΛ. ΚΑΘΗΓ. ΨΥΧΙΑΤΡΙΚΗΣ**

**ΕΡΕΥΝΗΤΙΚΟ ΠΡΟΓΡΑΜΜΑ**

**«ΑΝΑΓΚΕΣ ΠΛΗΡΟΦΟΡΗΣΗΣ, ΕΠΙΠΕΔΟ ΑΝΗΣΥΧΙΑΣ**

**ΚΑΙ ΑΝΤΙΚΤΥΠΟΣ ΤΗΣ ΝΕΑΣ ΓΡΙΠΠΗΣ ΣΤΗΝ ΨΥΧΙΚΗ ΚΑΤΑΠΟΝΗΣΗ**

**ΤΩΝ ΕΡΓΑΖΟΜΕΝΩΝ ΣΤΟ ΓΕΝΙΚΟ ΝΟΣΟΚΟΜΕΙΟ»**

**ΕΠΙΣΤ. ΥΠΕΥΘΥΝΟΣ: ΘΩΜΑΣ ΥΦΑΝΤΗΣ, ΑΝΑΠΛ. ΚΑΘΗΓ. ΨΥΧΙΑΤΡΙΚΗΣ**

Αγαπητοί συνεργάτες

Όπως όλοι μας γνωρίζουμε, από τον Απρίλιο 2009 ο ιός της γρίπης Α/Η1Ν1, γνωστής και ως «γρίπη των χοίρων», άρχισε να διασπείρεται στις περισσότερες χώρες του κόσμου, με αποτέλεσμα ο Παγκόσμιος Οργανισμός Υγείας να αυξήσει πρόσφατα το επίπεδο ετοιμότητας πανδημίας στο επίπεδο 6, που σημαίνει ότι επίκειται μια παγκόσμια πανδημία.

Η εξέλιξη αυτή έχει θέσει τα Νοσοκομεία της χώρας μας σε κατάσταση ετοιμότητας, ώστε να ανταπεξέλθουν στην αντιμετώπισης της νέας γρίπης. Καθώς πρόκειται για μείζον ζήτημα δημόσιας υγείας, είναι σημαντικό να γνωρίζουμε πόσο ανησυχούν, ποιες ανάγκες πληροφόρησης έχουν και πόσο επιδρά το όλο θέμα στην ψυχική καταπόνηση των εργαζομένων στο νοσοκομείο μας, εργαζόμενοι που σε τελευταία ανάλυση βρίσκονται στην πρώτη γραμμή αντιμετώπισης της νόσου.

Στενά συνδεδεμένη με όλο το προσωπικό του Νοσοκομείου μας, λόγω της φύσης των υπηρεσιών που παρέχει, η Μονάδα Συμβουλευτικής-Διασυνδετικής Ψυχιατρικής έχει σχεδιάσει μια έρευνα με στόχο την διερεύνηση των αναγκών πληροφόρησης, του επιπέδου ανησυχίας και του αντίκτυπου της νέας γρίπης στην ψυχική καταπόνηση του προσωπικού του Νοσοκομείου μας, ώστε να μπορέσουμε να εντοπίσουμε μερικούς ακόμη ασθενείς κρίκους στην αλυσίδα αντιμετώπισης της νόσου και να προτείνουμε τεκμηριωμένα μέτρα για την αρτιότερη κάλυψη των αναγκών του προσωπικού.

Για το σκοπό αυτό, σας ζητάμε να διαθέσετε λίγα λεπτά από τον χρόνο σας και να συμπληρώσετε το ερωτηματολόγιο που ακολουθεί, διαβεβαιώνοντάς σας πως η συμμετοχή σας στην έρευνα αυτή θα συμβάλλει σημαντικά στην αντιμετώπιση αρκετών από τα προβλήματα που αντιμετωπίζει το προσωπικό του Νοσοκομείου μας σε σχέση με τη νέα γρίπη.

Σας διαβεβαιώνουμε ότι οι απαντήσεις σας θα χρησιμοποιηθούν αποκλειστικά και μόνον για τους σκοπούς της συγκεκριμένης έρευνας και αποκλειστικά και μόνον από την συγκεκριμένη ερευνητική ομάδα, οι δε προσωπικές πληροφορίες θα κωδικοποιηθούν ώστε να είναι αδύνατη η διασταύρωση των προσωπικών στοιχείων με τις απαντήσεις σας, ενώ τα στοιχεία της ταυτότητάς σας θα χρησιμοποιηθούν μόνον για να επικοινωνήσουμε εκ νέου μαζί σας.

Θα είμαι προσωπικά στην διάθεσή σας για κάθε περαιτέρω πληροφορία.

Ευχαριστώντας σας εκ των προτέρων

Με τιμή

Θωμάς Υφαντής

Αναπληρωτής Καθηγητής Ψυχιατρικής

e-mail: [tyfantis@cc.uoi.gr](mailto:tyfantis@cc.uoi.gr)

**Έντυπο συγκατάθεσης**

1. Τα στοιχεία και τα προσωπικά δεδομένα που θα προκύψουν θα είναι αυστηρά απόρρητα.
2. Τα δεδομένα θα χρησιμοποιηθούν αποκλειστικά και μόνον για τους ερευνητικούς σκοπούς της συγκεκριμένης έρευνας.
3. Το όνομα και η διεύθυνσή σας θα κρατηθούν χωριστά από τα στοιχεία που θα προκύψουν από τις απαντήσεις σας, θα είναι κωδικοποιημένα και θα είναι γνωστά μόνον στον επιστημονικό υπεύθυνο της έρευνας.
4. Τα στοιχεία που θα προκύψουν θα αναλυθούν χωρίς το όνομά σας από τον ηλεκτρονικό υπολογιστή.
5. Το όνομα και η διεύθυνσή σας θα χρησιμοποιηθούν μόνο για να σας ξαναεπισκεφθούμε μετά από κάποιο χρονικό διάστημα. Μετά από αυτό, έντυπα που περιέχουν το όνομα και την διεύθυνσή σας θα καταστραφούν.

Εάν δέχεστε να συμμετάσχετε, παρακαλούμε σημειώστε τα στοιχεία που σας ζητούνται και υπογράψτε.

**ΣΥΜΦΩΝΙΑ ΣΥΜΜΕΤΟΧΗΣ ΣΤΗΝ ΕΡΕΥΝΑ**

**Ονοματεπώνυμο: …………………………………………………………………………………**

**Έχω πληροφορηθεί για την έρευνα του Πανεπιστημίου Ιωαννίνων ««ΑΝΑΓΚΕΣ ΠΛΗΡΟΦΟΡΗΣΗΣ, ΕΠΙΠΕΔΟ ΑΝΗΣΥΧΙΑΣ ΚΑΙ ΑΝΤΙΚΤΥΠΟΣ ΤΗΣ ΝΕΑΣ ΓΡΙΠΠΗΣ ΣΤΗΝ ΨΥΧΙΚΗ ΚΑΤΑΠΟΝΗΣΗ ΤΩΝ ΕΡΓΑΖΟΜΕΝΩΝ ΣΤΟ ΓΕΝΙΚΟ ΝΟΣΟΚΟΜΕΙΟ», τους σκοπούς της, την διαδικασία συμμετοχής και τον χρόνο που θα απαιτηθεί γι αυτό. Είχα σαφή και πλήρη εξήγηση της φύσης και του σκοπού της εξέτασης και του συγκεκριμένου λόγου για τον οποίο εξετάζομαι, καθώς και για το πώς θα χρησιμοποιηθούν τα αποτελέσματα που θα προκύψουν από την εξέτασή μου. Γνωρίζω, επιπλέον, ότι έχω δικαίωμα να ενημερωθώ για τα αποτελέσματα, καθώς και ότι έχω δικαίωμα να διακόψω την συμμετοχή μου στην εξέταση οποτεδήποτε και χωρίς κυρώσεις.**

**Ο κάτωθι υπογεγραμμένος συμφωνώ οικειοθελώς να συμμετάσχω στην έρευνα με βάση τις προϋποθέσεις που μου έχουν αναλυτικά επεξηγηθεί από τους συνεργάτες της ερευνητικής ομάδας.**

**Ημερομηνία: ………………………………………………………………………………….**

**Υπογραφή:……………………………………………………………………………………..**

**ΔΗΜΟΓΡΑΦΙΚΑ ΣΤΟΙΧΕΙΑ - ΓΕΝΙΚΕΣ ΠΛΗΡΟΦΟΡΙΕΣ**

Ημερομηνία ………………………………

Ονοματεπώνυμο ………………………………………………………………………………………………

Φύλο: Άνδρας 🞏 Γυναίκα 🞏

Ηλικία: ………………

| Οικογενειακή Κατάσταση: | Έγγαμος/η.….……..  Άγαμος/η.………….. Διαζευγμένος/η……Συζώ. ………………..Χήρος/α………….…… | 🞏  🞏  🞏  🞏  🞏 |
| --- | --- | --- |

Έχετε παιδιά; Ναι 🞏 Όχι 🞏

Εάν έχετε παιδιά, τι ηλικία έχουν; ………………………………………………………………..

Εκπαίδευση:

| Αναλφάβητος/η………………………………………...  Απόφοιτος δημοτικού …………………………….…  Απόφοιτος Γυμνασίου ………………………………  Απόφοιτος Λυκείου ………………………………..…  Φοιτητής/-τρια …….……………………….……….…  Απόφοιτος ΤΕΙ ………………………………………….  Απόφοιτος ΑΕΙ ……………………………………….… | 🞏  🞏  🞏  🞏  🞏  🞏  🞏 |
| --- | --- |

Σε ποια Κλινική ή Τμήμα του Νοσοκομείου εργάζεστε; ……………………………………………….

Είστε:

| Νοσηλευτικό Προσωπικό ………….  Ιατρός ……………………………….……..  Ειδικευόμενος/η Ιατρός ……………  Φοιτητής/τρια Ιατρικής……………..  Διοικητικό Προσωπικό……………….  Τεχνικό Προσωπικό ………………....  Προσωπικό Κυλικείου ……………….  Καθαρίστρια……………………………….  Άλλο …………………………………….…… | 🞏  🞏  🞏  🞏  🞏  🞏  🞏  🞏  🞏 |
| --- | --- |

| Μένετε: | Σε χωριό…………………………………………………..… Σε κωμόπολη μέχρι 10.000 κατοίκους………  Σε πόλη από 10.000-150.000 κατοίκους….. | 🞏  🞏  🞏 |
| --- | --- | --- |

**ΕΡΩΤΗΣΕΙΣ ΣΧΕΤΙΚΑ ΜΕ ΤΗ ΝΕΑ ΓΡΙΠΠΗ**

1. **Σας ανησυχεί η επιδημία της νέας γρίπης**; ΝΑΙ 🞏 ΟΧΙ 🞏

Αν απαντήσατε ΝΑΙ : Η ανησυχία αφορά (σημειώστε όλες τις απαντήσεις που σας αντιπροσωπεύουν):

🞏 Την επικινδυνότητα της νόσου

🞏 Τον κίνδυνο μετάδοσής της στους οικείους σας

🞏 Τον φόβο απομόνωσής σας από τον οικογενειακό και κοινωνικό σας περίγυρο

🞏 Τις επιπτώσεις της νόσου (σε περίπτωση που νοσήσετε) στη λειτουργικότητά σας

(οικογενειακές, εργασιακές, κοινωνικές υποχρεώσεις).

1. **Εάν βαθμολογούσατε τον βαθμό ανησυχίας σας, τι βαθμό θα βάζατε;** (κυκλώστε έναν αριθμό)

Πολύ λίγο ανησυχώ 1 2 3 4 5 6 7 8 9 Ανησυχώ πάρα πολύ

1. **Πιστεύω ότι έχω ενημερωθεί επαρκώς σχετικά με:** (κυκλώστε έναν αριθμό)

α. Τα **συμπτώματα** της νόσου:

Διαφωνώ απολύτως 1 2 3 4 5 6 7 8 9 Συμφωνώ απολύτως

β. Την **πρόγνωση** της νόσου:

Διαφωνώ απολύτως 1 2 3 4 5 6 7 8 9 Συμφωνώ απολύτως

γ. Την **θεραπεία** της νόσου:

Διαφωνώ απολύτως 1 2 3 4 5 6 7 8 9 Συμφωνώ απολύτως

ε. Τον **τρόπο μετάδοσης** της νόσου:

Διαφωνώ απολύτως 1 2 3 4 5 6 7 8 9 Συμφωνώ απολύτως

στ. Τα συνιστώμενα **μέτρα πρόληψης**:

Διαφωνώ απολύτως 1 2 3 4 5 6 7 8 9 Συμφωνώ απολύτως

1. **Μερικοί άνθρωποι, αφού πάρουν όλες τις πληροφορίες που τους είναι απαραίτητες σχετικά με μια νόσο από την οποία ενδέχεται να αρρωστήσουν και τη θεραπεία της, προτιμούν να μη μαθαίνουν περισσότερες λεπτομέρειες, ενώ άλλοι προτιμούν να παίρνουν επιπρόσθετες πληροφορίες. Παρακαλούμε βάλτε σε κύκλο την πρόταση που ταιριάζει καλύτερα στις δικές σας προτιμήσεις.**

Προτιμώ να παίρνω:

| **1**  Όχι περισσότερες πληροφορίες από αυτές που μου χρειάζονται | **2**  Λίγες περισσότερες πληροφορίες | **3**  Μερικές περισσότερες πληροφορίες | **4**  Πολύ περισσότερες πληροφορίες | **5**  Όσο το δυνατόν περισσότερες πληροφορίες |
| --- | --- | --- | --- | --- |

1. **Πιστεύω ότι η υπηρεσία μου παρείχε στο προσωπικό σαφή πληροφόρηση σχετικά με την πανδημία** (κυκλώστε έναν αριθμό)

Διαφωνώ απολύτως 1 2 3 4 5 6 7 8 9 Συμφωνώ απολύτως

1. **Θεωρώ ότι το τμήμα/υπηρεσία όπου εργάζομαι είναι επαρκώς προετοιμασμένο για την πανδημία**  (κυκλώστε έναν αριθμό)

Διαφωνώ απολύτως 1 2 3 4 5 6 7 8 9 Συμφωνώ απολύτως

1. **Πόσο μεγάλη πιστεύετε ότι είναι η πιθανότητα να μολυνθείτε από τον νέο ιό;**

Πολύ μικρή 1 2 3 4 5 6 7 8 9 Πολύ μεγάλη

1. **Πόσο αποτελεσματικά θεωρείτε ότι είναι τα συνιστώμενα μέτρα πρόληψης**;

Καθόλου αποτελεσματικά 1 2 3 4 5 6 7 8 9 Πολύ αποτελεσματικά

1. **Εφαρμόζετε τα συνιστώμενα μέτρα πρόληψης**:

α. Στο χώρο του νοσοκομείου:….. ΝΑΙ 🞏 ……… ΟΧΙ 🞏

β. Εκτός του νοσοκομείου:……… ΝΑΙ 🞏 ……… ΟΧΙ 🞏

1. **Πιστεύω ότι μια πιθανή προσβολή μου από τη νέα γρίπη θα είχε σοβαρές συνέπειες για την υγεία μου.**

Διαφωνώ απολύτως 1 2 3 4 5 6 7 8 9 Συμφωνώ απολύτως

1. **Πιστεύω ότι η νέα γρίπη είναι δύσκολο να θεραπευτεί**

Διαφωνώ απολύτως 1 2 3 4 5 6 7 8 9 Συμφωνώ απολύτως

1. **Έχετε έλθει σε επαφή με άτομο που νοσούσε**;……………….…..….. ΝΑΙ 🞏 …. ΟΧΙ 🞏
2. **Έχετε νοσήσει από τη νέα γρίπη** ; …………………………………..... ΝΑΙ 🞏 …. ΟΧΙ 🞏
3. **Έχει νοσήσει κάποιο μέλος της οικογένειάς σας από τη νέα γρίπη**; … ΝΑΙ 🞏 …. ΟΧΙ 🞏
4. **Έχετε περιορίσει τις κοινωνικές σας επαφές γιατί ο χώρος εργασίας σας θεωρείται «επικίνδυνος»;** ………………….… ΝΑΙ 🞏 ………… ΟΧΙ 🞏
5. **Πιστεύετε ότι οι οικείοι σας αποφεύγουν την επαφή μαζί σας διότι εργάζεστε σε περιβάλλον υψηλού κινδύνου**; ……ΝΑΙ 🞏 ……… ΟΧΙ 🞏
6. **Τον τελευταίο καιρό ανησυχείτε τόσο σχετικά με τη νέα γρίπη που θα παίρνατε ακόμη και άδεια για να αποφύγετε να νοσήσετε**; …… ΝΑΙ 🞏……… ΟΧΙ 🞏
7. **Εάν το Νοσοκομείο χρειαζόταν οπωσδήποτε τις υπηρεσίες σας σε μια κατάσταση αυξημένου κινδύνου μετάδοσης της νόσου, πόσο πιθανόν θα ήταν να προσπαθήσετε να το αποφύγετε;**

Πολύ πιθανό 1 2 3 4 5 6 7 8 9 Καθόλου πιθανό

1. **Νομίζω ότι θα ήταν σημαντικό να υπάρχει κάποιος φορέας που να μπορεί να προσφέρει ψυχολογική υποστήριξη σε θέματα σχετικά με τις ανησυχίες μου για τη νέα γρίπη;**

Διαφωνώ απολύτως 1 2 3 4 5 6 7 8 9 Συμφωνώ απολύτως

1. **Εάν βαθμολογούσατε τον βαθμό ικανοποίησης από την εργασία σας, τι βαθμό θα βάζατε**;

Καθόλου ικανοποιημένος/η 1 2 3 4 5 6 7 8 9 Πολύ ικανοποιημένος/η

**ΕΡΩΤΗΜΑΤΟΛΟΓΙΟ ΓΕΝΙΚΗΣ ΥΓΕΙΑΣ (GHQ-28)**

Παρακαλούμε διαβάστε τα παρακάτω προσεκτικά. Θα θέλαμε να ξέρουμε αν είχατε κάποια ενοχλήματα, και γενικά πως ήταν η υγεία σας τις **τελευταίες εβδομάδες**. Παρακαλούμε να απαντήσετε σε **όλες** τις παρακάτω ερωτήσεις, σημειώνοντας την απάντηση που νομίζετε ότι σας ταιριάζει καλύτερα. Προσέξτε μόνο, θα θέλαμε να ξέρουμε τα **τωρινά και πρόσφατα** ενοχλήματα σας και όχι αυτά που είχατε στο παρελθόν. Έχει σημασία να προσπαθήσετε να απαντήσετε σε **όλες** τις ερωτήσεις.

**ΤΟΝ ΤΕΛΕΥΤΑΙΟ ΚΑΙΡΟ:**

| **Αισθανόσαστε**  **εντελώς καλά,**  **απόλυτα υγιής;** | Καλύτερα απ’ ό,τι συνήθως  **1** | Το ίδιο όπως συνήθως  **2** | Χειρότερα απ’ ότι  Συνήθως  **3** | Πολύ χειρότερα  απ’ ό,τι συνήθως  **4** |
| --- | --- | --- | --- | --- |
|  |  |  |  |  |
|  | Καθόλου | Όχι περισσότερο  απ’ ό,τι συνήθως | Μάλλον περισσότερο  απ’ ό,τι συνήθως | Πολύ περισσότερο  απ’ ό,τι συνήθως |
| **Νοιώθατε την**  **ανάγκη για κάποιο**  **δυναμωτικό;** | **1** | **2** | **3** | **4** |
| **Νοιώθατε**  **εξαντλημένος/η**  **& κακοδιάθετος/η;** | **1** | **2** | **3** | **4** |
| **Είχατε αισθανθεί**  **πως είσαστε άρρωστος/η;** | **1** | **2** | **3** | **4** |
| **Είχατε καθόλου**  **πόνους στο κεφάλι;** | **1** | **2** | **3** | **4** |
| **Νοιώθατε σφίξιμο ή βάρος στο κεφάλι;** | **1** | **2** | **3** | **4** |
| **Είχατε περιόδους**  **που αισθανόσαστε**  **κρυάδες ή εξάψεις;** | **1** | **2** | **3** | **4** |
| **Έχετε συχνά χάσει τον ύπνο σας επειδή ήσαστε ανήσυχος/η;** | **1** | **2** | **3** | **4** |
| **Έχετε δυσκολία να συνεχίσετε τον ύπνο σας χωρίς διακοπές από την στιγμή που θα αποκοιμηθείτε;** | **1** | **2** | **3** | **4** |
| **Έχετε αισθανθεί ότι βρισκόσαστε συνεχώς σε**  **υπερένταση;** | **1** | **2** | **3** | **4** |
| **Είσαστε οξύθυμος/η και αρπαζόσαστε**  **εύκολα;** | **1** | **2** | **3** | **4** |
| **Νοιώθατε φόβο**  **ή πανικό χωρίς**  **σοβαρό λόγο;** | **1** | **2** | **3** | **4** |
| **Αισθανόσαστε πως σας έχουν καταπλακώσει**  **οι καθημερινές**  **ασχολίες;** | **1** | **2** | **3** | **4** |
| **Αισθανόσαστε συνεχώς νευρικός/ή και σε υπερδιέγερση;** | Καλύτερα απ’ ό,τι συνήθως  **1** | Το ίδιο όπως συνήθως  **2** | Χειρότερα απ’ ότι  Συνήθως  **3** | Πολύ χειρότερα  απ’ ό,τι συνήθως  **4** |
| **Έχετε καταφέρει να είσαστε δραστήριος/α**  **και πάντα**  **απασχολημένος/η;** | Περισσότερο  απ’ ό,τι συνήθως  **1** | Το ίδιο όπως  συνήθως  **2** | Μάλλον λιγότερο  απ’ ό,τι συνήθως  **3** | Πολύ λιγότερο  απ’ ό,τι συνήθως  **4** |

**ΤΟΝ ΤΕΛΕΥΤΑΙΟ ΚΑΙΡΟ:**

| **Σας παίρνει**  **περισσότερο**  **χρόνο να κάνετε**  **τις δουλειές σας** | Γρηγορότερα  απ’ ό,τι συνήθως  **1** | Το ίδιο όπως  Συνήθως  **2** | Περισσότερο  απ’ ό,τι συνήθως  **3** | Πολύ περισσότερο  απ’ ό,τι συνήθως  **4** |
| --- | --- | --- | --- | --- |
| **Αισθανόσαστε**  **ότι σε γενικές**  **γραμμές τα**  **καταφέρνατε καλά;** | Καλύτερα απ’  ό,τι συνήθως  **1** | Περίπου το ίδιο  **2** | Χειρότερα  απ’ ό,τι συνήθως  **3** | Πολύ χειρότερα  απ’ ό,τι συνήθως  **4** |
| **Είσαστε**  **ικανοποιημένος/η**  **με τον τρόπο που**  **κάνατε τις**  **δουλειές σας;** | Περισσότερο  Ικανοποιημένος/η  **1** | Περίπου το ίδιο  όπως συνήθως  **2** | Λιγότερο  ικανοποιημένος/η  απ’ ό,τι συνήθως  **3** | Πολύ λιγότερο  ικανοποιημένος/η  **4** |
| **Έχετε αισθανθεί**  **ότι παίζετε χρήσιμο ρόλο σε ότι συμβαίνει**  **γύρω σας;** | Περισσότερο  απ’ ό,τι συνήθως  **1** | Το ίδιο όπως  συνήθως  **2** | Λιγότερο χρήσιμο  απ’ ό,τι συνήθως  **3** | Πολύ λιγότερο  χρήσιμο απ’ ό,τι  συνήθως  **4** |
| **Αισθανόσαστε**  **ικανός/ή να**  **παίρνετε αποφάσεις**  **για διάφορα θέματα;** | Περισσότερο  απ’ ό,τι συνήθως  **1** | Το ίδιο όπως  συνήθως  **2** | Λιγότερο απ’ ό,τι  συνήθως  **3** | Πολύ λιγότερο  απ’ ό,τι συνήθως  **4** |
| **Μπορούσατε να χαρείτε τις συνηθισμένες**  **καθημερινές**  **δραστηριότητες σας;** | Περισσότερο  απ’ ό,τι συνήθως  **1** | Το ίδιο όπως  συνήθως  **2** | Λιγότερο απ’ ό,τι  συνήθως  **3** | Πολύ λιγότερο  απ’ ό,τι συνήθως  **4** |
| **Σκεφτόσαστε**  **πως δεν αξίζετε**  **τίποτα;** | Καθόλου  **1** | Όχι περισσότερο  απ’ ό,τι συνήθως  **2** | Μάλλον περισσότερο  απ’ ό,τι συνήθως  **3** | Πολύ περισσότερο  απ’ ό,τι συνήθως  **4** |
| **Αισθανόσαστε**  **πως η ζωή είναι**  **χωρίς καμιά**  **ελπίδα;** | Καθόλου  **1** | Όχι περισσότερο  απ’ ό,τι συνήθως  **2** | Μάλλον περισσότερο  απ’ ό,τι συνήθως  **3** | Πολύ περισσότερο  απ’ ό,τι συνήθως  **4** |
| **Αισθανόσαστε**  **πως δεν αξίζει**  **κανείς να ζει;** | Καθόλου  **1** | Όχι περισσότερο  απ’ ό,τι συνήθως  **2** | Μάλλον περισσότερο  απ’ ό,τι συνήθως  **3** | Πολύ περισσότερο  απ’ ό,τι συνήθως  **4** |
| **Σας πέρασε από**  **το μυαλό η**  **πιθανότητα να**  **δώσετε τέλος**  **στη ζωή σας;** | Σίγουρα όχι  **1** | Δεν νομίζω  **2** | Πέρασε από  το μυαλό μου  **3** | Σίγουρα το  Σκέφτηκα  **4** |
| **Βρίσκατε μερικές φορές ότι δεν μπορούσατε να**  **κάνετε τίποτα γιατί τα νεύρα σας ήταν σε**  **άσχημη κατάσταση;** | Καθόλου  **1** | Όχι περισσότερο  απ’ ό,τι συνήθως  **2** | Μάλλον περισσότερο  απ’ ό,τι συνήθως  **3** | Πολύ περισσότερο  απ’ ό,τι συνήθως  **4** |
| **Πιάνατε τον εαυτό σας να εύχεται να**  **είσαστε πεθαμένος/η**  **και να είχατε ξεμπερδέψει απ’ όλα;** | Καθόλου  **1** | Όχι περισσότερο  απ’ ό,τι συνήθως  **2** | Μάλλον περισσότερο  απ’ ό,τι συνήθως  **3** | Πολύ περισσότερο  απ’ ό,τι συνήθως  **4** |
| **Βρίσκετε πως η ιδέα να δώσετε τέλος στη ζωή σας ερχόταν**  **συνεχώς στο μυαλό σας;** | Καθόλου  **1** | Δεν νομίζω  **2** | Πέρασε από  το μυαλό μου  **3** | Σίγουρα ναι  **4** |
