# Supplementary material for: General hospital staff worries, perceived sufficiency of information and associated psychological distress during the A/H1N1 influenza pandemic
Source: BMC Infect Dis. 2010 Nov 9;10:322. doi: 10.1186/1471-2334-10-322 (PMC2990753; doi:10.1186/1471-2334-10-322)
Supplement: Additional file 2 — Questionnaire for assessing hospital staff worries and perceived sufficiency of information during the A/H1N1 influenza pandemic (English version). This file contains the English translation of the questionnaire administered to assess hospital staff worries and perceived sufficiency of information during the A/H1N1 influenza pandemic. [file 1471-2334-10-322-S2.PDF]

Questionnaire for assessing general hospital staff's worries, perceived sufficiency of information and attitudes towards the A/H1N1 pandemic

1. **Do you worry about the swine flu pandemic?** YES ☐ NO ☐

   If YES, you mostly worry about:
   (Please fill-in all possible answers that might represent your worry)

   ☐ The disease's dangerousness
   ☐ The risk for family and relatives' to be infected
   ☐ Isolation from family and/or social environment
   ☐ The consequences on your functional ability regarding family, work or social relationships (in case you would be infected)

2. **How would you score your degree of worry?** (circle a number)

   I'm very little worried 1 2 3 4 5 6 7 8 9 I'm very much worried

3. **I believe that I have heard sufficient information about:** (circle a number)

   a. Swine flu symptoms: .... I strongly disagree 1 2 3 4 5 6 7 8 9 I strongly agree
   b. Swine flu prognosis: ………… I strongly disagree 1 2 3 4 5 6 7 8 9 I strongly agree
   c. Swine flu treatment:…………. I strongly disagree 1 2 3 4 5 6 7 8 9 I strongly agree
   d. Swine flu contamination route: .I strongly disagree 1 2 3 4 5 6 7 8 9 I strongly agree
   e. Swine flu preventive measures: I strongly disagree 1 2 3 4 5 6 7 8 9 I strongly agree

4. **After they have all the information they need about an infectious illness that might suffer and its treatment, some people prefer not to get any more details, and others prefer to get additional information. Please circle the sentence better reflecting your preference:**

   I prefer to get:

**1**	**2**	**3**	**4**	**5**
No more details than needed	A few more details	Some more details	Many more details	As many more details as possible

5. **I believe that my department provided clear information about the swine flu pandemic** (circle a number)

   I strongly disagree 1 2 3 4 5 6 7 8 9 I strongly agree

6. **I feel that my ward/department is well prepared for the swine flu pandemic** (circle a number)

   I strongly disagree 1 2 3 4 5 6 7 8 9 I strongly agree

7. **I believe that the risk for being infected by the A/H1N1 virus is:**

Very low 1 2 3 4 5 6 7 8 9 Very high

8. **How effective do you believe the recommended preventive measures are?**

Not at all effective 1 2 3 4 5 6 7 8 9 Very effective

9. **Do you implement the recommended preventive measures:**

In the hospital premises: YES ☐ NO ☐

Outside the hospital premises YES ☐ NO ☐

10. **I think that being infected with the swine flu would have major consequences on my health**

I strongly disagree 1 2 3 4 5 6 7 8 9 I strongly agree

11. **I believe that the infection is difficult to be treated**

I strongly disagree 1 2 3 4 5 6 7 8 9 I strongly agree

12. **Have you been in contact with an infected person?** ……………... YES ☐ NO ☐

13. **Have you been infected with the swine flu? ....** YES ☐ NO ☐

14. **Has a member of your family been infected with the swine flu? …** YES ☐ NO ☐

15. **I have restricted my social contacts because my work environment is considered "dangerous" ……………………………** YES ☐ NO ☐

16. **I feel that my family members and friends avoid contacts with me, because I work in a "high-risk" environment …………...** YES ☐ NO ☐

17. **Lately I am so concerned about the swine flu, that I would take a leave to avoid going to work …………………………………..**YES ☐ NO ☐

18. **In an emergency situation due to the swine flu pandemic, how much possible would be to avoid your duties?**

Highly possible 1 2 3 4 5 6 7 8 9 Not at all possible

19. **I think it would be important if there was a service offering psychological support regarding my concerns about the swine flu pandemic**

I strongly disagree 1 2 3 4 5 6 7 8 9 I strongly agree

20. **How would you score the degree you are satisfied by your work?**

Not at all satisfied 1 2 3 4 5 6 7 8 9 Very much satisfied
